# 1319. Microbiology and Epidemiology of Pediatric Ventriculoperitoneal Shunt Infections

**DOI:** 10.1093/ofid/ofad500.1158

**Published:** 2023-11-27

**Authors:** Kevin M Claunch, Daniel Adams, Sarah Deperrior, Michael Rajnik

**Affiliations:** Walter Reed National Military Medical Center , Bethesda, Virginia; Naval Medical Center Portsmouth, Portsmouth, Virginia; Defense Centers for Public Health, Portsmouth, Virginia; Uniformed Services University of the Health Sciences, Bethesda, MD

## Abstract

**Background:**

Infections complicate approximately 10% of ventriculoperitoneal (VP) shunts in children, with considerable associated morbidity and mortality. Recent guidelines for empiric treatment of VP shunt infections recommend use of vancomycin combined with an anti-pseudomonal beta-lactam, but are based on low-quality evidence. We aimed to further characterize and update the microbiology of pediatric VP shunt infections using the U.S. Military Health System database.

**Methods:**

Department of Defense beneficiaries less than 22 years-of-age admitted for VP shunt infection between Oct 1, 2008 and Sep 30, 2019 were included. VP shunt infection was defined using a combination of ICD-9-CM and ICD-10-CM codes, CPT codes for removal or replacement of a shunt (Table 1), and positive cultures from a normally sterile site (cerebrospinal fluid, blood, catheter tip, and peritoneum). Demographic data on age, sex, and year of admission were collected for each patient, as was microbiologic data including organism and specimen source.

**Figure:**
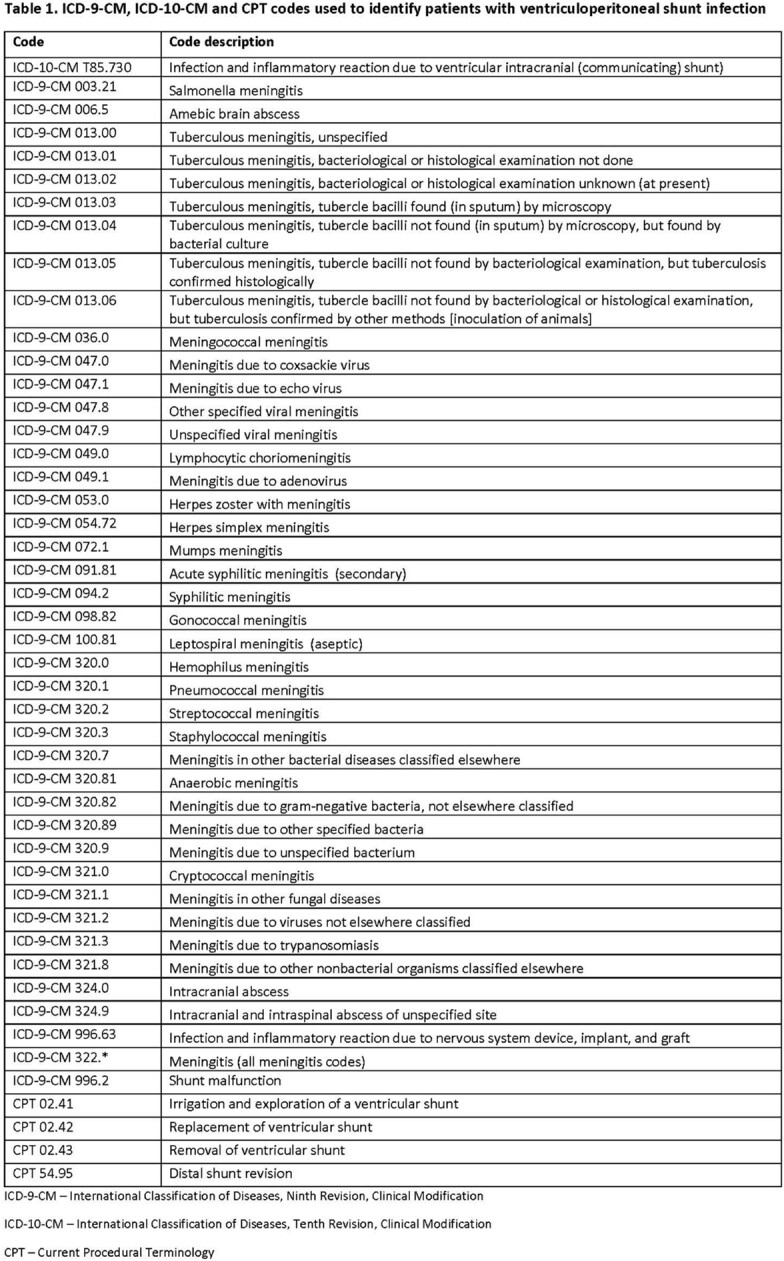

**Results:**

There were 32 qualifying admissions among 30 patients for VP shunt infection over the 10-year study period, with no discernable year-to-year trend identified. Half of all VP shunt infections occurred in patients less than 12 months of age (Table 2). Sixty-one unique positive cultures were identified among the 30 patients, 38 (62%) from CSF samples, and the remaining 23 (38%) from blood, peritoneum, and catheter tips (Figure 1). Coagulase-negative Staphylococcus (28%), S*taphylococcus aureus* (15%), and *Enterococcus* spp. (10%) were the leading causative pathogens (Figure 2). Enteric gram-negative rods accounted for 21% of positive cultures. *Pseudomonas* spp. were rarely isolated (3%), however, strict anaerobic pathogens made up 8% of positive cultures.

**Figure:**
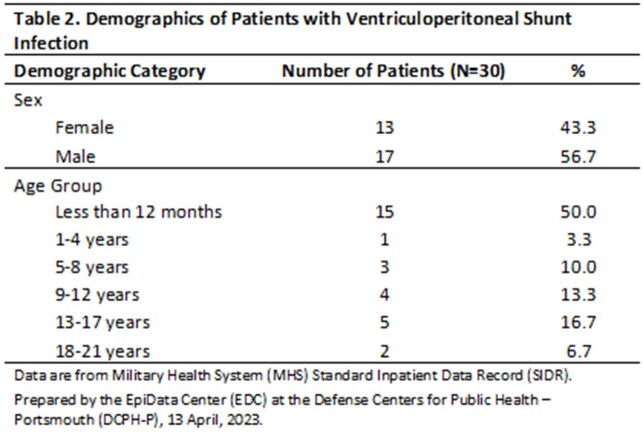

**Figure:**
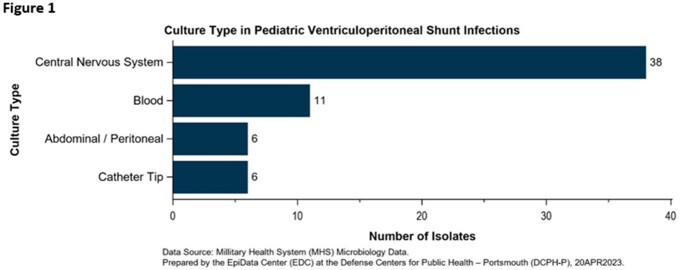

**Figure:**
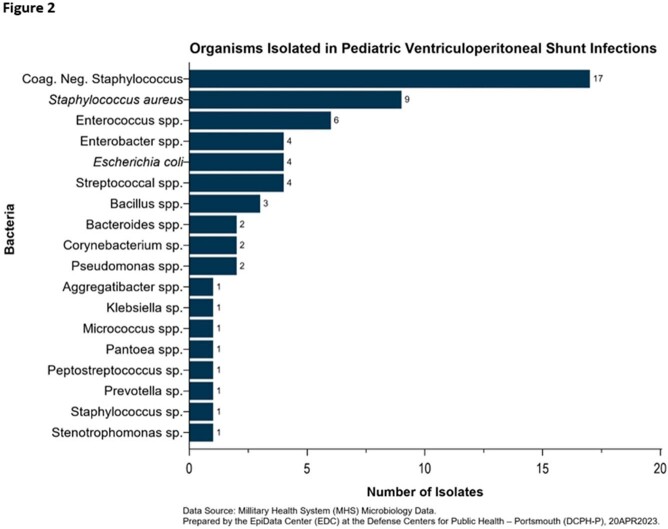

**Conclusion:**

Empiric antibiotic treatment of VP shunt infections in children should cover gram-positive cocci and enteric gram-negative rods but need not cover *Pseudomonas* spp. Existing guidelines do not recommend anaerobic coverage, however, these data indicate empiric anaerobic coverage is prudent, especially when an intra-abdominal VP shunt infection is suspected.

**Disclosures:**

**All Authors**: No reported disclosures

